# Adenoids in the Naso-Pharynx

**Published:** 1896-02

**Authors:** Edward J. Bermingham

**Affiliations:** Surgeon in Chief; New York


THE
### Southern Medical Record.

A MONTHLY JOURNAL OF MEDICINE AND SURGERY.

VOL. XXVI. ATLANTA, GA., FEBRUARY, 1896. No. 2.

### Original Articles.

### ADENOIDS IN THE NASO-PHARYNX.

A CLINICAL LECTURE DELIVERED AT THE NEW YORK THROAT AND NOSE HOSPITAL.

BY EDWARD J. BERMINGHAM, A. M., M. D.

Surgeon in Chief.

GENTLEMEN.—The first case which I present to you to-day, a boy two years old, was brought to the hospital two weeks ago suffering from incomplete nasal obstruction, snoring, catarrh, and great restlessness and difficulty of breathing during the night. Adenoids had been removed by means of the finger nail by a specialist in this city about two months previously, and although this had given temporary relief, the child had been as bad as ever for a month before he came here. Anterior rhinoscopic examination at that time revealed no obstruction, but the faucial tonsils were enlarged, and digital examination showed the naso-pharynx to be filled with adenoids. The tonsils were guillotined, and the naso-pharynx cleaned out with curette and forceps, without an anæsthetic. The child breathed freely at once, and the mother reports that he has breathed and slept quietly since.

The next case is a boy five years old, who came to the hospital a month ago complaining of deafness. Adenoids were found covering both Eustachian openings and the faucial tonsils were enlarged. Nasal respiration was impeded, particularly at night, when the patient would frequently wake crying and, sitting up in bed, would struggle for breath. Under chloroform, the tonsils were guillotined and the naso-pharynx cleared by curette and forceps. Hearing was restored in less than a week, and the child has slept quietly since the operation.

The third case is this boy, eleven years old, sent to us by his physician with the statement that his nose has probably been broken and that he cannot breathe through it. He presents an unusually bright appearance, owing to the fact that the disease has existed but a short time. The nostrils, however, as you may observe, do not move with respiration and the mouth is constantly open. He is slightly deaf, but the symptom for which he seeks relief is nasal obstruction. He complains of nothing else. An anterior rhinoscopic examination reveals no obstruction whatever, and but slight hypertrophic rhinitis. Examination of the throat shows normal tonsils and slight follicular pharyngitis. But he cannot get the slightest particle of air through the nose. We now pass the forefinger of the right hand up behind the soft palate into the naso-pharynx and find it completely filled with a soft, irregular growth which has very appropriately been described as feeling like a "bunch of worms."

Just beneath the mucous membrane in the pharyngeal vault occupying the roof and posterior wall of the pharynx, lining its lateral walls and surrounding the orifices of the Eustachian tubes, may be found a mass of small, closed, ductless glands. This is known as the pharyngeal or Luschka's tonsil. It is similar in structure to the faucial tonsils, and, in a normal condition is about a quarter of an inch in thickness. This mass often becomes permanently diseased and hypertrophied, and the pathological condition is variously known as adenoids, adenoid vegetation, hypertrophy of the pharyngeal or of Luschka's tonsil, and lymphoid hypertrophy at the pharyngeal vault. This diseased condition was first described by Czermak, in 1860, who was followed by Loewenberg, in 1865; but it is to Wilhelm Meyer, of Copenhagen, that we owe our chief knowledge of this condition. In 1870 he described the disease so fully as to leave hardly anything further to be said in regard to it at the present day.

This hypertrophy is usually found in children under fifteen years of age, most commonly between three and twelve, and generally accompanies the conditions known as follicular pharyngitis and hypertrophy of the faucial tonsils. In fact, in almost every case of chronically enlarged faucial tonsils in children we will, upon examination with the finger, be able to detect more or less hypertrophy of the pharyngeal tonsil.

Enlargement of the pharyngeal tonsil is, in my opinion, caused principally by nasal stenosis, whether due to permanent encroachment upon the nasal cavities by organized growths, or by repeated congestions and thickenings of the mucous membrane lining the cavities, and caused by simple, acute, and chronic rhinitis so common in the children of the poor. Whenever we have obstruction to the nasal respiration from any cause, we have diminished air tension behind the obstruction, each act of respiration is accompanied by a distension of the blood vessels behind the seat of obstruction, which, in time, produces hypertrophy. It is in this way that most, if not all, hypertrophies in the upper air passages are produced.

You have, in the patients before you, the typical physiognomy of those suffering from marked nasal obstruction, whether that obstruction exists in the anterior or posterior nares, or at any intervening point. It is, in fact, the expression of countenance of the habitual mouth breather. Hypertrophy of the pharyngeal tonsil always causes some degree of obstruction of the posterior nares, and the patient is compelled to breathe through the mouth. The nonuse of the nostrils in respiration causes atrophy of the dilators of the alæ nasi, and the nostrils become small and flattened, and the nose has a pinched appearance, which, with the open mouth gives the child a stupid expression. Although the naso-pharynx has been cleared in two of these cases, this expression still remains, owing to the establishment of the habit of mouth breathing, which it will take some time to break the little patients of.

In the majority of cases of adenoids, we also find another marked symptom when we speak to the child and find that we have to repeat the question and raise the voice before we receive an answer. Indeed, it is this symptom of deafnesss for which we are consulted in fully one-half the cases of adenoids. It is always more or less marked and is caused either by catarrh of the Eustachian tube or middle ear from extension of the catarrhal condition from the naso-pharynx, or by closure of the Eustachian openings by the adenoid growths. Other marked symptoms in this condition are the impairment of the olfactory sense, and the effects on articulation. The latter is particularly well marked, and is due both to the closure of the nose and the filling up of the nasopharyngeal space. There is consequently an absence of sonorous vibration and the tones are deadened. P or M is pronounced B, and T or N has the sound of D. The remaining symptoms consist of nasal and postnasal catarrh, snoring, restlessness, cough, and sometimes laryngismus, stridulus and convulsions.

We shall now proceed to operate upon this case. It is best to use an anæsthetic in older children, but in those under two or two and a half years of age, I prefer to operate without an anæsthetic. In the case of an infant, it is best to roll the patient in a sheet, confining the arms, and have it held upright on the knee of an assistant, the operator sitting squarely in front of the patient. Entrust the mouth gag to an assistant, whose sole business will be to attend to it and keep it in place between the molar teeth. Introduce the Gottstein curette behind the palate into the pharyngeal vault and sweep the cutting edge over the surface from the interior portion of the vault, backwards, and then downwards. This can readily be accomplished by elevating the handle of the instrument, and it will remove the bulk of the adenoid tissue. Now introduce the forefinger of the left hand and hold a Hooper forceps in the right hand, behind the palate, and as each particle of lymphoid hypertrophy is felt with the left forefinger as a guide, seize and remove it with the forceps. The child will frequently seem on the point of strangling, when the finger and forceps should be quickly removed, to be reintroduced and the process continued. This should be kept up until not a particle of hypertrophied tissue remains.

In older children, as in the case before us, it is best to give an anæsthetic, and to operate with the patient lying down and the head inclined to one side. If the tonsils are hypertrophied, a portion of them should first be removed, so as to give us more room to work in. Then use the Gottstein curette as described above, and finish with the Quinlan forceps. Always use the forefinger of the left hand for a guide and to determine when the pharyngeal vault is cleared. The bleeding is sometimes free, but will generally cease in a few minutes. The after treatment consists in cleansing the nose and naso-pharynx several times daily with one part of thymenol and four of tepid water.

Now, one word in regard to the return of this affection after operation. We are repeatedly assured that if we are careful to remove every particle of hypertrophied tisssue, the disease will not return, but that it is not only likely, but very certain to do so if any is left behind. This statement is not in accordance with my experience. I find that in those cases in which the hypertrophy recurs, there exists nasal stenosis in front of the naso-pharynx, either due to causes which may be removed by surgical means, or to repeated and neglected attacks of rhinitis. If permanent stenosis of the nasal cavities exists, correct it by surgical means before finally dismissing your patient, and caution the parents in regard to the prompt treatment and relief of every "cold in the head," which the child contracts. Do this, and I am convinced you will not have recurring adenoids after operation.

---

CANCER OF THE BREAST.—A skin incision, embracing a liberal area around the nipple, and running across the axilla to the point of insertion of the tendon of the pectoralis major muscle, is made. A second incision is made at right angles to the one just described, running to the junction of the middle and outer thirds of the clavicle. After the skin flaps are reflected, the tendons of insertion of the pectoralis major and minor muscles are divided, and these muscles, the axillary, subclavicular and infraclavicular fat and lymphatics, and the diseased breast, are removed in one mass. The muscles are separated from their points of origin, and the new growth is not cut into during the operation. The vessels entering the pectoralis major muscles are clamped before they are cut. The wound is sutured as far as possible and axillary drainage employed.—DOCTOR W. MEYER, in *Medical Record.*

